# Beyond Discrete Choices – Investigating the Effectiveness of a Proximity Nudge With Multiple Alternative Options

**DOI:** 10.3389/fpsyg.2020.01211

**Published:** 2020-06-10

**Authors:** Laurens C. van Gestel, Marieke A. Adriaanse, Denise T.D. de Ridder

**Affiliations:** Department of Social, Health, and Organisational Psychology, Utrecht University, Utrecht, Netherlands

**Keywords:** nudging, choice architecture, food choice, decision complexity, choice overload

## Abstract

Nudges are defined as small adjustments in the choice architecture that stimulate desirable behavior. Nudging techniques can be used as a promising policy tool, but research has hardly systematically taken into account the complexity of the situation in which nudges have been implemented. In the current studies, we investigated the effectiveness of a proximity nudge on food choice in a realistic situation with multiple options in the immediate surroundings of the target option. In two studies, we presented participants from a community sample with an assortment of either three or nine different types of chocolate. For half of the participants, the target chocolate was placed most proximally on a table. Across two studies, we demonstrated that the proximity nudge was effective in stimulating the choice for a specific piece of chocolate in a simple and more complex situation. Results were further qualified by Bayesian analyses, which revealed most support for the hypothesis that the proximity effect existed in both the conditions with three and nine options, regardless of the number of options in the choice set. Results imply that the proximity effect can remain robust in realistic situations that include multiple options in the immediate environment to choose from.

## Introduction

In recent years, more and more research has been devoted to interventions called nudges: small adjustments in the choice architecture that stimulate a specific choice option, without forbidding alternative options or financially interfering with them (Thaler and Sunstein, 2008). While many studies have shown promising results, surprisingly little attention has been paid to the hypothesized working mechanisms or boundary conditions of their effectiveness. A recent scoping review revealed that the majority of published studies aimed to demonstrate the effectiveness of nudges in a particular setting (Szaszi et al., 2018). Oftentimes, these settings are rather simplified settings in which a decision maker has to decide between choosing one out of two alternatives. For example, in the realm of health behavior, studies have focused on the likelihood of consumption of one available snack (Maas et al., 2012) or on consumption of one out of two alternatives (Privitera and Zuraikat, 2014). In other behavioral fields, such as sustainable behavior, similar simplified settings have been used to study the effectiveness of nudges, such that participants are given the choice between one green and one gray energy plan (Vetter and Kutzner, 2016). In reality, however, people often face a complex choice of choosing between multiple products for consumption or multiple energy plans. This is reflected in field studies on nudging (Cadario and Chandon, 2019), where there are multiple alternative options in the wider environment, but a systematic comparison of nudge effectiveness with multiple alternative options in the immediate environment is missing. Therefore, in order to move the field forward, we suggest that more systematic research is needed, where nudges are studied in more realistic, complex situations that involve multiple options.

People in Western societies have an ever-increasing number of options to choose from a wide variety of settings. To illustrate, many supermarkets have increased their assortments such that consumers nowadays have up to an entire isle with different varieties of cereal, supposedly enabling them to choose their most preferred option. Yet, many studies have documented limitations to human decision making in terms of being unable to consider a wide variety of options (Schwartz, 2004). Investigating the effectiveness of nudges in complex contexts that involve a multitude of options is thus important as these types of choices are the choices that people struggle with particularly, and which they encounter increasingly often. Therefore, in the current set of studies, we aim to investigate whether the effectiveness of a nudge is dependent on the number of alternatives to choose from. We do so by investigating the effectiveness of a proximity nudge that employs the distance of the desired alternative as a nudge to encourage a specific choice.

### Nudging and Choice Architecture Interventions

Nudges are defined as small adjustments in the choice architecture that stimulate a specific choice. They make strategic use of biases or rules of thumb that guide people’s behavior, such as the status quo bias (Samuelson and Zeckhauser, 1988) or the social proof heuristic (Cialdini, 2009). Inherent to the definition of nudges is that alternative options remain readily available and are not forbidden or financially made less attractive. Nudging as such is thought to be an umbrella term for a wide variety of interventions that alter cues in the physical and/or social environment to promote a choice that is deemed desirable by the choice architect. Hence, many different types of nudges have been applied across various behavioral fields. Prototypical examples range from defaults that create an opt-out system as opposed to an opt-in system (e.g., Madrian and Shea, 2001; Johnson and Goldstein, 2003; Pitchert and Katsikopoulos, 2008) to making desirable behavior more salient (e.g., Wilson et al., 2016) or placing the desirable option more proximally (Maas et al., 2012; Hunter et al., 2018). These and other types of nudges have been applied in a wide variety of domains, including health, sustainable, and prosocial behavior.

Although there are notable differences between these nudging interventions, they all share the principle of making it easier to perform the desirable behavior, which is the cornerstone of the idea behind nudging. Building on research on human decision making and information processing that highlights two systems of thinking (intuitive vs. reflective; see Evans, 2008, for an overview), nudges are assumed to align with automatic processes (Marteau et al., 2011). That is, nudges aim to steer behavior without taxing cognitive resources by making strategic use of automatic tendencies. This implies that nudging interventions are in principle not dependent on cognitive capacity in order to be effective. Preliminary evidence seems to be in line with this proposition, as it has been shown that increasing the distance toward a bowl filled with M&M’s decreases the likelihood of taking any snacks, regardless of cognitive resources (trait or state; Hunter et al., 2018). Similarly, in a study investigating the use of sugar tongs as a means of increasing the effort needed to acquire a certain snack, it was found that the effect on intake is not dependent on the availability of cognitive resources (state; Brunner, 2013). At the same time, there is suggestive evidence indicating that nudges are more effective in the crucial circumstances when deliberate reasoning is inhibited due to reduced self-control capacity (Salmon et al., 2014). Nevertheless, these studies altogether seem to imply that nudges capitalize on effortless processes involved in the decision making process.

### Proximity

One of the most typically studied nudges in the domain of food choice relies on the proximity effect: the phenomenon that people are most likely to choose an option that is presented most proximally to them. For example, it has been shown that placing an unhealthy snack further away from a participant decreases both the likelihood of consuming that snack as well as the amount of consumption (Maas et al., 2012). The effect on likelihood of consumption was recently replicated among participants who did not move the bowl filled with snacks such that the distance manipulation remained intact (Hunter et al., 2018). A recent review of positional influences further revealed a positive effect in 16 out of 18 studies (Bucher et al., 2016). Yet, when more options are on offer, results thus far seem mixed. One study provided suggestive evidence for the existence of a proximity effect in a so-called competitive food environment, where people were offered the choice between two snack options that differed in healthiness (Privitera and Zuraikat, 2014). In this study, it was shown that, even though participants reported a higher liking for the unhealthy snack (popcorn) than for the healthy snack (apple slices), participants consumed most of the snack that was placed most proximally to them. A recent experiment, which employed a similar design with one healthy and one unhealthy snack, however, revealed different results. This study showed that the likelihood of consuming the unhealthy snack (M&M’s) was affected by its own proximity such that it was more often consumed when placed proximally. The likelihood of consuming the healthy snack (raisins), however, was neither affected by its own proximity or by the proximity of the unhealthy snack (Hunter et al., 2019). Altogether, these studies point out that there is a need to study the proximity effect in more complex situations.

In this light, it is striking to observe that the current evidence base of nudging effectiveness lacks a systematic approach in regard to the number of options in the immediate environment. On the one hand, nudges have been studied extensively in field studies, where there are numerous options in the wider environment to choose from (Cadario and Chandon, 2019). To illustrate, a recent field study on the effectiveness of a repositioning nudge was conducted in a kiosk that included up to 179 food products (Van Gestel et al., 2018). These types of field studies have been conducted in a wide variety of settings with varying levels of complexity in the wider environment, but a systematic investigation of the possible impact that the level of complexity may have is missing. On the other hand, experimental research is mostly limited to studies on binary choices of either or not choosing, or of choosing a desirable (e.g., sustainable, healthy, etc.) option over an undesirable (e.g., unsustainable, unhealthy, etc.) option. To illustrate, studies on proximity have typically investigated the likelihood of consumption of one snack when there is only one snack on offer (Maas et al., 2012; Hunter et al., 2018) or consumption of one out of two options (Privitera and Zuraikat, 2014; Hunter et al., 2019). These experimental studies thus often lack a more realistic complex situation with multiple alternative options in the immediate proximity of the target option. Therefore, to advance the knowledge base and effective implementation of the nudges in daily practice, it is essential to acquire a further understanding of the conditions under which the effectiveness remains existent. In particular, a stronger focus on the number and type of alternatives is called for, since there may be aspects inherent to the choice set at hand that complicate the decision making process.

### Choice Sets

Research in behavioral judgment and decision making has highlighted the importance of considering the wider choice context in nudging research. Research on choice overload, for example, has suggested that, even though people tend to cherish the idea of freedom of choice, the more options there are to choose from, the more complicated the decision becomes. Consequently, decision outcomes may not always be optimal and may reflect subjectively or objectively suboptimal decisions (Schwartz, 2004). And while the necessary precedents as well as the behavioral and affective consequences of choice overload are debated upon (Scheibehenne et al., 2010; Chernev et al., 2015; McShane and Böckenholt, 2018), the phenomenon at least illustrates the importance of considering the number and type of alternative options when stimulating one particular option. In fact, for the study of nudges, it is important to consider the wider choice architecture when introducing one small adjustment to it. Moreover, given that nudges are thought to capitalize on effortless processes, it is important to examine whether the effects remain robust in exactly those circumstances that require more effort to reach a decision. Our goal in the current studies is not to manipulate choice overload as such, but rather to systematically investigate whether the number of options in a choice set impacts nudge effectiveness.

### The Current Studies

In the current studies, we aim to systematically investigate whether the proximity effect on the likelihood of choosing a certain option for consumption remains robust in situations where there are more alternatives to choose from. Having more options to choose from increases the complexity of the decision and may spread attention over these alternatives, thereby making it a more challenging environment for the nudge to stimulate desirable behavior. Yet, it is often argued that nudges capitalize on effortless processes, which could imply that nudges remain effective in exactly these more challenging situations where more effort is called for in order to reach a decision. In two studies, we let participants pick a piece of chocolate out of an assortment of either three or nine alternatives with equal utility. For half of the participants, we restructured the assortment structure so that the target chocolate was placed more proximally to the participant. Across these studies, we focused on the likelihood of choosing a particular option for consumption, rather than the likelihood of consumption or the amount of consumption. Inspired by research on choice overload, we also measured subjective difficulty and experienced doubt while making the choice. No *a priori* hypotheses were formulated for these measures.

## Study 1

### Materials and Methods

#### Participants and Design

Study 1 was conducted as part of an alumni event of Utrecht University. In total, 134 (85 women, 48 men, for one participant gender was not recorded) participants, with an average age of 41.86 (*SD* = 18.22), participated in the study. The study used an experimental 2 (number of options: 3 vs. 9) × 2 (nudge: absent vs. present) between-subjects design. The main dependent variable was whether or not participants chose the target chocolate (which was positioned most proximally in the nudge conditions).

#### Procedure

Upon arrival at the alumni event, people were invited to partake in the study. If participants indicated to be willing to participate in the study, they received a questionnaire, which was color coded with a red, blue, green, or yellow header in order to randomize participants over the four conditions. After having filled out the questionnaire, participants could hand it in at the location with the matching color code (i.e., participants who filled out a questionnaire with a green header were requested to hand it in at the table with a green label). When participants handed in their questionnaire, they were informed that they could choose a piece of chocolate as a reward, after which we would ask them a few more questions about their choice. The presentation of these chocolates differed between the four conditions such that there were either three or nine options and such that one of the options was placed most proximally or not. After having picked a piece of chocolate, participants were asked to fill out a follow-up questionnaire, which, upon completion, was stapled to the original questionnaire by one of the experimenters.

#### Materials

##### Primary Questionnaire

Participants initially received the primary questionnaire, which was a Dutch version of the Rational Experiential Inventory – short form (Norris et al., 1998; Witteman et al., 2009). This questionnaire was used for exploratory purposes, which were beyond the purpose of the current study, and simultaneously served as the starting point of the study.

##### Choice of Chocolates

Participants were ostensibly rewarded for filling out the primary questionnaire with a piece of chocolate of their choice. In line with previous studies on choice overload (e.g., Iyengar and Lepper, 2000), we took care in controlling for prior preferences. The chocolates used in the current study were Quality Street chocolates, which are relatively unfamiliar amongst the Dutch population. We chose these chocolates as our choice set because there are a large enough variety of options. Thereby, the choice was reduced to a decision between different chocolates of different shapes and colors. All chocolates were sorted and each type was presented in a separate bowl. Throughout the study, careful attention was paid to the number of chocolates presented in each bowl, and the bowls were frequently refilled so as not to install an implicit norm indicating a popular piece of chocolate (Prinsen et al., 2013).

**Figure 1:**
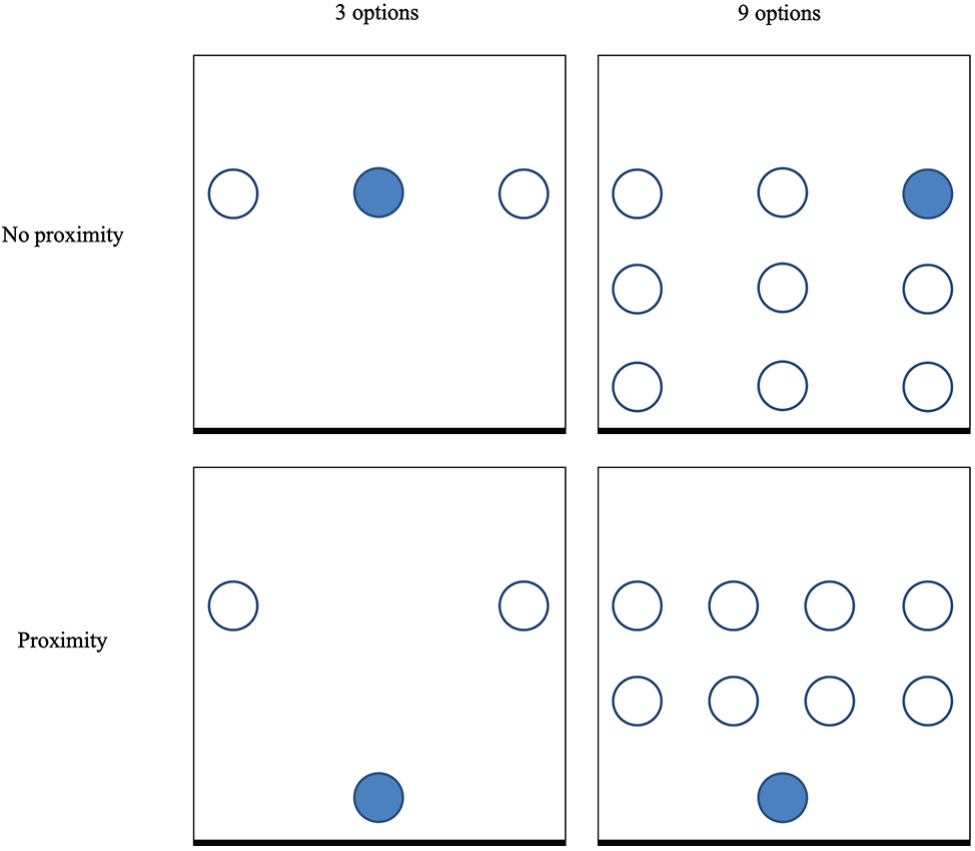
Schematic overview of the set-up in Study 1. The squares represent a table, and the thicker lines at the bottom represent the side of the table from which participants would approach the set-up. The circles represent bowls with chocolate, and the filled circle represents the bowl with the target chocolate.

The number of options as well as the presentation of these options differed in the four conditions. In the conditions with three options, participants could choose between three alternatives presented in three separate bowls, while in the conditions with nine options, participants could choose between nine alternatives presented in nine separate bowls. The three types of chocolates that were used in the conditions with three options were different from the nine types of chocolates that were used in the conditions with nine options. Within those conditions, the target chocolate remained the same. In the conditions without the nudge, the options were presented as three in a row, or as a matrix of three by three. In the conditions with the nudge, one of the options was presented more proximally. Importantly, although the bowl filled with the target chocolate was placed more proximally, all alternative options remained within arm’s reach (see Figure 1 for a graphic overview of the set-up).

##### Follow-up Questionnaire

The follow-up questionnaire, which was administered after participants had chosen their piece of chocolate, contained the main variables of interest. First, participants were presented with pictures of the chocolates and were asked to encircle the chocolate they had just chosen. Next, we asked participants questions about their experience with the decision making process, focusing on the subjective difficulty of the choice (“How difficult was it to choose from the different options?”), satisfaction with the choice (“How satisfied are you with your choice?”)^1^, and experienced doubt (“To what extent did you doubt your choice?”). These questions were asked on 7-point Likert scales, ranging from 1 (*not at all*) to 7 (*Very much*). Next, participants were asked if they were familiar with Quality Street chocolates (*yes*/*no*), and whether they had an *a priori* preference (*yes*/*no*). Finally, participants reported their age and gender.

#### Data Analysis Plan

We used both frequentist and Bayesian statistics in R. The main frequentist analysis was a stepwise logistic regression on the dependent variable whether or not participants had chosen the target chocolate. In the first block, only a main effect of the nudge was investigated. In the second block, a main effect of the number of options was added. In the third and final block, the interaction effect of the proximity nudge and number of options was added. Follow-up analyses consisted of ANOVAs with subjective difficulty and experienced doubt as dependent variables.

Besides, we conducted a logistic regression with Bayesian statistics using the R package Bain (Gu et al., 2018). By conducting these analyses, we were able to account for base rate likelihoods (33% chance of choosing a particular piece of chocolate if presented with three options vs. 11% chance of choosing a particular piece of chocolate if presented with nine options). We did this by multiplying the observed probabilities of choosing the target chocolate in the conditions with three options with a weight of three, while multiplying the observed probabilities of choosing the target chocolate in the conditions with nine options with a weight of nine. Moreover, we used these additional analyses to evaluate the evidential base for three separate, informative, hypotheses. The statistical hypotheses that were evaluated and compared were as follows: (1) the nudge is ineffective no matter the number of options and there is no interaction effect, (2) the nudge is effective in both the condition with three and nine options, but there is no interaction effect, and (3) the nudge is effective in both the condition with three and nine options, and there is an interaction effect such that the nudge is more effective in the condition with nine options than in the condition with three options.

### Results

#### Descriptives

Table 1 presents the descriptive statistics of all variables under study.

**Table 1:** Descriptive statistics for the sample characteristics and main variables of interest by condition for Study 1.

Characteristics	Group				All participants
	Three options, no proximity	Three options, proximity	Nine options, no proximity	Nine options, proximity	
*n*	37	34	34	29	134
Age (*M*(*SD*))	41.65 (19.61)	42.62 (18.57)	41.32 (17.12)	41.86 (18.16)	41.86 (18.22)
Gender (%(*n*))
Male	45.95 (17)	41.18 (14)	23.53 (8)	31.03 (9)	35.82 (48)
Female	54.05 (20)	58.82 (20)	73.53 (25)	68.97 (20)	63.43 (85)
NA	0.00 (0)	0.00 (0)	2.94 (1)	0.00 (0)	0.75 (1)
Difficulty (*M*(*SD*))	2.38 (1.55)	2.59 (1.48)	2.97 (1.71)	3.10 (1.74)	2.74 (1.63)
Doubt (*M*(*SD*))	2.78 (1.69)	2.65 (1.69)	2.94 (1.65)	2.79 (1.42)	2.79 (1.61)
Satisfaction (*M*(*SD*))	5.03 (1.69)	5.12 (1.30)	5.09 (1.63)	5.24 (1.64)	5.11 (1.56)
Familiar (%(*n*))
Yes	64.86 (24)	50.00 (17)	44.12 (15)	37.93 (11)	50.00 (67)
No	35.14 (13)	50.00 (17)	55.88 (19)	62.07 (18)	50.00 (67)
Preference (%(*n*))
Yes	10.81 (4)	2.94 (1)	23.53 (8)	27.59 (8)	15.67 (21)
No	89.19 (33)	97.06 (33)	76.47 (26)	72.41 (21)	84.33 (113)
Target chocolate chosen (%(*n*))	37.84 (14)	64.71 (22)	14.71 (5)	24.14 (7)	35.82 (48)

#### Randomization Check

Across the four conditions, participants did not differ from each other in age (*F* < 1, *p* = 0.993) or gender (*χ*^2^(3) = 4.27, *p* = 0.234), indicating successful randomization of participants. Exactly half of the participants indicated to be familiar with Quality Street chocolates. A large majority of 113 participants indicated to have had no prior preference when selecting a piece of chocolate.

#### Main Analyses

In order to analyze the main research question, a logistic regression model was built using the stepwise method. In step 1, the main effect of the nudge was added. This model provided significant model fit, *χ*^2^(1) = 5.42, *p* = 0.020, and revealed a main effect of the nudge, *b* = 0.85 (*SE* = 0.37), *p* = 0.021, *OR* = 2.33, 95% *CI* [1.14, 4.87], implying that participants were more than twice as likely to choose the target chocolate when this option was positioned proximally than when it was not positioned proximally. In step 2, the main effect of the number of options was added. Adding this independent variable significantly improved model fit, *χ*^2^(1) = 15.38, *p* < 0.001, so that the model again fitted the data well, *χ*^2^(2) = 20.80, *p* < 0.001. Adding the number of options to the model did not change significance for the main effect of the nudge, *b* = 0.93 (*SE* = 0.39), *p* = 0.019, *OR* = 2.53, 95% *CI* [1.18, 5.57]. The model also revealed a main effect of the number of options, *b* = −1.53 (*SE* = 0.41), *p* < 0.001, *OR* = 0.22, 95% *CI* [0.09, 0.47], indicating that participants were about 4.5 times more likely to choose the target chocolate when offered three options than when offered nine options. This ratio was to be expected given differing base rates. In step 3, the interaction effect of the nudge and the number of options was added. Adding this interaction effect did not improve model fit, *χ*^2^(1) = 0.36, *p* = 0.550. In line with that, the interaction effect did not prove to be significant, *b* = −0.50 (*SE* = 0.82), *p* = 0.548, *OR* = 0.61, 95% *CI* [0.12, 3.12].

Excluding those who indicated to have had a prior preference (*N* = 21) did not change the pattern of results, although the significant main effect of the nudge became marginally significant (*p* = 0.056 in block 1, *p* = 0.068 in block 2). Similarly, including age and gender as covariates did not change the pattern or significance of the main results nor did these covariates have a significant effect on the likelihood of choosing the target chocolate.

#### Bayesian Analyses

Bayesian analyses revealed relatively little evidence for the first hypothesis, stating the absence of any nudge or interaction effect compared with its complement, *BF*_1*c*_ = 2.68. Contrarily, relative to its complement, there seems to be strong evidence for the effect of the nudge in both the condition with three and nine options, *BF*_2*c*_ = 15.75. Finally, relative to its complement, there seems to be some positive evidence for both an effective nudge and an interaction effect with the number of options, *BF*_3*c*_ = 4.44. Relative to each other, positive evidence for the second evaluated hypothesis – that the nudge is effective in both the condition with three and nine options and that this effect is not moderated by the number of options – was found, *BF*_21_ = 5.87 and *BF*_23_ = 5.85.

#### Follow-up Analyses

Follow-up analyses revealed no main effect of the nudge (*p* = 0.543) on subjective difficulty nor an interaction effect with the number of options (*p* = 0.891). However, results did reveal a marginally significant main effect of the number of options on subjective difficulty, *F*(1, 130) = 3.89, *p* = 0.051, *η_p_*^2^ = 0.03, such that participants in the condition with three options found it less difficult (*M* = 2.48, *SD* = 1.51) than participants in the condition with nine options (*M* = 3.03, *SD* = 1.71) to reach a decision. Regarding experienced doubt, no main or interaction effects were found (all *ps* > 0.591).

### Discussion

Study 1 revealed suggestive evidence for the effectiveness of the nudge in both the condition with three and nine options. In this study, we found strongest evidence for the hypothesis that this effect is present regardless of the number of options to choose from. Follow-up analyses suggested that it was easier for participants to choose a piece of chocolate when presented with few rather than a lot of alternatives, but this effect was only marginally significant. Overall, Study 1 revealed interesting trends but was rather underpowered. Therefore, we decided to conceptually replicate Study 1 with a larger sample and an improved set-up of the experiment.

## Study 2

### Materials and Methods

#### Participants and Design

Study 2 was conducted at the Dutch National Health Fair. Based on the results of Study 1, the required sample size was calculated from analytical expressions for the relation between power and sample size in Moerbeek and Maas (2005), while ignoring the multilevel data structure. For this sample size calculation, we used the odds ratio for the main effect of the nudge in Study 1 (i.e., *OR* = 2.53) and the main effect of the number of options (i.e., *OR* = 0.22). These expressions assume equal sample sizes in each of the four groups of the factorial design. As these sample sizes varied slightly in Study 1, the power is likely to be overestimated by a few percent. The analysis revealed that, in order to replicate a statistically significant main effect of the nudge with 80% power, at least 194 participants needed to be recruited. An additional sensitivity analysis further revealed that with 410 participants we would be able to achieve 80% power with effect sizes that would deviate with 25% from those found in Study 1 [i.e., a 25% smaller odds ratio for the main effect of the nudge (*OR* = 1.90), and a 25% larger odds ratio for the main effect of the number of options (*OR* = 0.16)].

In total, we had 4 days of data collection which resulted in 412 (353 women, 58 men, for one participant gender was not recorded) participants, with an average age of 44.63 (*SD* = 17.56). The study used a quasi-experimental 2 (number of options: 3 vs. 9) × 2 (nudge: absent vs. present) between-subjects design. The main dependent variable was whether or not participants chose the target chocolate (which was positioned most proximally in the nudge conditions).

#### Procedure

The National Health Fair lasted 4 days. Each day was divided in a morning session and an afternoon session and in each of the sessions one of the conditions was set up. The order in which the conditions were taking place was counterbalanced, so that each condition was run in one morning session and one afternoon session, as well as once during a workday and once during a day in the weekend.

Participants were welcomed at the stand and were asked if they wanted to participate in a study on food choices. After giving informed consent, the study started with an unrelated questionnaire for which they would be rewarded with a piece of chocolate. Just as in Study 1, participants were provided with a follow-up questionnaire in which we asked questions about the chocolate they had just chosen. After having filled out all questionnaires, participants were thanked for their participation.

#### Materials

##### Primary Questionnaire

At the start of the study, participants received a bogus questionnaire that we used to enforce the cover story that the chocolate was a reward.

##### Choice of Chocolates

**Figure 2:**
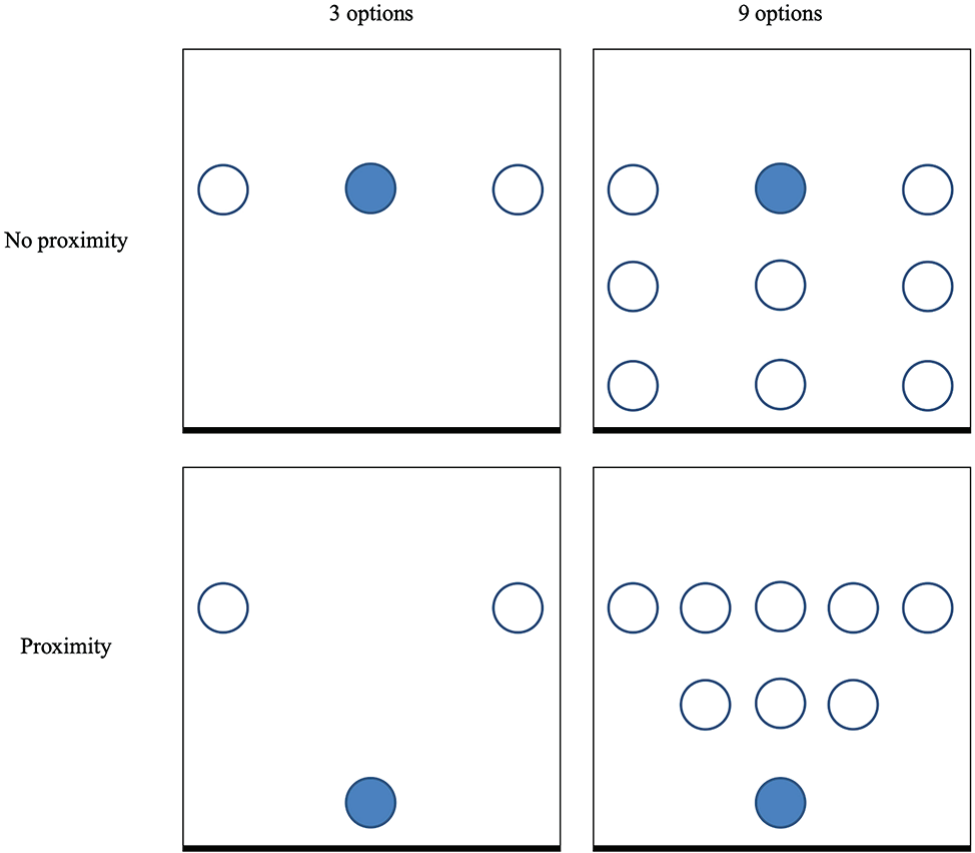
Schematic overview of the set-up in Study 2. The squares represent a table, and the thicker lines at the bottom represent the side of the table from which participants would approach the set-up. The circles represent bowls with chocolate, and the filled circle represents the bowl with the target chocolate.

Participants were ostensibly rewarded for filling out the primary questionnaire with a piece of chocolate of their choice. We used the same chocolates as in Study 1. Study 2 largely resembled Study 1 in the design, but a different set-up of the presentation of the chocolates was used in the conditions with nine options. First, in the condition without the nudge, the bowl with the target chocolate was placed centrally rather than in the corner. Second, in the condition with the proximity nudge, a similar kind of triangular structure as in the condition with three options was used (See Figure 2 for a graphic overview of the set-up).

##### Follow-up Questionnaire

We used the same follow-up questionnaire as in Study 1^2^.

#### Data Analysis Plan

We used the same data analysis plan as in Study 1 and again conducted both frequentist and Bayesian analyses, this time in a more confirmatory manner.

### Results

#### Descriptives

Table 2 presents the descriptive statistics of all variables under study.

**Table 2:** Descriptive statistics for the sample characteristics and main variables of interest by condition for Study 2.

Characteristics	Group				All participants
	Three options, no proximity	Three options, proximity	Nine options, no proximity	Nine options, proximity	
*n*	83	95	124	110	412
Age (*M*(*SD*))	45.95 (18.70)	43.96 (18.37)	43.66 (17.14)	45.32 (16.53)	44.63 (17.56)
Gender (%(*n*))
Male	14.46 (12)	13.68 (13)	15.32 (19)	12.73 (14)	14.08 (58)
Female	84.34 (70)	86.32 (82)	84.68 (105)	87.27 (96)	85.68 (353)
NA	1.20 (1)	0.00 (0)	0.00 (0)	0.00 (0)	0.24 (1)
Difficulty (*M*(*SD*))	2.89 (1.74)	2.40 (1.67)	2.50 (1.57)	2.82 (1.89)	2.64 (1.72)
Doubt (*M*(*SD*))	3.04 (1.76)	2.52 (1.62)	2.69 (1.76)	2.79 (1.78)	2.75 (1.74)
Satisfaction (*M*(*SD*))	4.22 (2.04)	4.68 (1.82)	4.93 (1.85)	5.02 (1.73)	4.75 (1.90)
Familiar (%(*n*))
Yes	55.42 (46)	58.95 (56)	54.84 (68)	56.36 (62)	42.48 (175)
No	42.17 (35)	38.95 (37)	44.35 (55)	43.64 (48)	56.31 (232)
NA	2.41 (2)	2.11 (2)	0.81 (1)	0.00 (0)	1.21 (5)
Preference (%(*n*))
Yes	16.87 (14)	18.95 (18)	22.58 (28)	20.91 (23)	20.15 (83)
No	81.93 (68)	82.05 (77)	75.81 (94)	78.18 (86)	78.88 (325)
NA	1.20 (1)	0.00 (0)	1.61 (2)	0.91 (1)	0.97 (4)
Target chocolate chosen (%(*n*))	38.55 (32)	54.74 (52)	14.52 (18)	28.18 (31)	32.28 (133)

#### Randomization Check

Across the four conditions, participants did not differ from each other in age (*F* < 1, *p* = 0.766) or gender (*χ*^2^(3) = 0.36, *p* = 0.949), indicating successful randomization of participants. Over half of the participants (*N* = 232) indicated to be familiar with Quality Street chocolates. A large majority of 325 participants indicated to have had no prior preference when selecting a piece of chocolate.

#### Main Analyses

In order to analyze the main research question, a logistic regression model was built using the stepwise method. In step 1, the main effect of the nudge was added. This model provided significant model fit, *χ*^2^(1) = 12.19, *p* < 0.001, and revealed a main effect of the nudge, *b* = 0.74 (*SE* = 0.22), *p* < 0.001, *OR* = 2.11, 95% *CI* [1.38, 3.23], implying that participants were more than twice as likely to choose the target chocolate when this option was positioned proximally than when it was not positioned proximally. In step 2, the main effect of the number of options was added. Adding this independent variable significantly improved model fit, *χ*^2^(1) = 29.57, *p* < 0.001, so that the model again fitted the data well, *χ*^2^(2) = 41.76, *p* < 0.001. Adding the number of options to the model did not change significance for the main effect of the nudge, *b* = 0.72 (*SE* = 0.22), *p* = 0.001, *OR* = 2.06, 95% *CI* [1.33, 3.21]. The model also revealed a main effect of the number of options, *b* = −1.19 (*SE* = 0.22), *p* < 0.001, *OR* = 0.31, 95% *CI* [0.20, 0.47], indicating that participants were more than three times more likely to choose the target chocolate when offered three options than when offered nine options. Again, this ratio was to be expected given differing base rates. In step 3, the interaction effect of the nudge and the number of options was added. Adding this interaction effect did not improve model fit, *χ*^2^(1) = 0.19, *p* = 0.665. In line with that, the interaction effect did not prove to be significant, *b* = 0.19 (*SE* = 0.45), *p* = 0.665, *OR* = 1.22, 95% *CI* [0.50, 2.96].

Excluding those who had indicated to have a prior preference (*N* = 83) did not change the pattern or significance of the main results. Similarly, including age and gender as covariates did not change the pattern or significance of the main results nor did these covariates have a significant effect on the likelihood of choosing the target chocolate.

#### Bayesian Analyses

Bayesian analyses revealed little evidence for the first hypothesis stating the absence of any nudge or interaction effect compared with its complement, *BF*_1*c*_ = 0.69. Contrarily, relative to its complement, there seems to be relatively strong evidence for the hypothesis stating that the nudge is effective in both the condition with three and nine options, *BF*_2*c*_ = 11.00. Finally, relative to its complement, there seems to be strong evidence of both an effective nudge and an interaction effect with the number of options, *BF*_3*c*_ = 41.41.

Relative to each other, results showed strong positive evidence for the second evaluated hypothesis – that the nudge is effective in both the condition with three and nine options and that this effect is not moderated by the number of options – relative to the first evaluated hypothesis which specified the absence of a nudge and interaction effect, *BF*_21_ = 16.03. Similarly, results showed relatively strong positive evidence for the third evaluated hypothesis – that the nudge is effective in both the condition with three and nine options and that this effect is moderated by the number of options – relative to the first evaluated hypothesis, *BF*_31_ = 6.98. Lastly, there seems to be slightly more support for the second hypothesis in comparison to the third hypothesis, but the Bayes factor could not substantially differentiate between the two, *BF*_23_ = 2.30. Altogether, the data imply that the proximity nudge was effective in both conditions, with a remaining possibility that the nudge effect was most pronounced in the condition with nine options.

#### Follow-up Analyses

Follow-up analyses revealed no main effect of the nudge (*p* = 0.559) or number of options (*p* = 0.876) on subjective difficulty, but did reveal a significant interaction effect, *F*(1, 408) = 5.64, *p* = 0.018, *η_p_*^2^ = 0.014. *Post hoc* comparisons revealed that in the condition with three options, participants experienced the decision as easier when the nudge was present (*M* = 2.39, *SD* = 1.66) than when the nudge was absent (*M* = 2.89, *SD* = 1.74), *p* = 0.049. In the condition with nine options, there was no difference between the two groups in subjective difficulty (*p* = 0.175). Regarding experienced doubt, there was no main effect of the nudge (*p* = 0.194) nor a main effect of the number of options (*p* = 0.891). There was, however, a marginally significant interaction effect, *F*(1, 404) = 3.10, *p* = 0.079, *η_p_*^2^ = 0.01. *Post hoc* comparisons revealed that in the condition with three options, participants experienced less doubt when the nudge was present (*M* = 2.51, *SD* = 1.62) than when the nudge was absent (*M* = 3.04, *SD* = 1.76), *p* = 0.043. In the condition with nine options, there was no difference between the two groups in subjective difficulty (*p* = 0.727). Altogether, given the range of *p*, these exploratory analyses did not reveal robust evidence for any effects on subjective difficulty or experienced doubt.

### Discussion

Study 2 replicated the main results of Study 1 and revealed strong evidence for the effectiveness of the proximity nudge on the likelihood of choosing the target chocolate regardless the number of options involved. Follow-up analyses provided suggestive trends indicating decreased difficulty and experienced doubt due to the nudge in the condition with three options, but did not find a similar trend in the condition with nine options.

## General Discussion

Over the last decade, research on nudging as a promising novel technique for promoting desirable behavior has emerged. Considerable efforts have been made in establishing the evidence base of certain nudges in certain settings, but a systematic investigation of the effectiveness of nudges in settings that vary in complexity has largely been missing. While research in behavioral judgment and decision making has highlighted the importance of taking the characteristics of a choice set into account (Schwartz, 2004), this has so far not been embedded in research on nudging and the proximity effect. On the one hand, field studies have investigated the effectiveness of several nudges in wider environments that contain numerous options (Cadario and Chandon, 2019) but lack a systematic interpretation of the complexity of that environment. On the other hand, most empirical research on nudging have been dedicated to investigations of choosing one particular option or not (Maas et al., 2012; Hunter et al., 2018), or of choosing that option over another option (Privitera and Zuraikat, 2014; Hunter et al., 2019). Yet, it is important to advance the field by creating a thorough understanding of what works, but also of when and under what circumstances these nudges work in more complex, realistic, settings with alternative options in the immediate surroundings. In the current set of studies, we therefore made a systematic comparison of the effectiveness of the proximity nudge with choice sets consisting of three or nine alternatives.

Across two studies with community samples, we found support for the effectiveness of the proximity nudge on food choice in a real-life simple choice context as well as a real-life complex choice context involving multiple options. Results showed that participants were more than twice as likely to select the target chocolate when the bowl was placed proximally to the participant, thus revealing strong support for the effectiveness of a proximity nudge. Frequentist statistics further revealed that this effect of the proximity nudge was not moderated by the number of options in the choice set. In both studies, strongest evidence was found for the hypothesis that the nudge is effective in both the condition with three and nine options, without interaction with the number of options in the choice set. However, in Study 2, some support was also found for the possibility that the effect is even more pronounced in the condition with nine options. Follow-up analyses revealed suggestive trends regarding post-decision evaluations of experienced doubt and subjective difficulty, but did not reveal robust effects across the two studies.

In the current studies, we used choice sets that consisted of either three or nine options as a first examination of the robustness of the proximity effect on food choice with differing number of options. Results demonstrated that proximity remained effective in stimulating the selection of a specific option across these choice sets, implying that nudges can remain effective behavior change tools in more complex situations involving a multitude of alternative options. This is important for the ecological validity of studies on nudging, since people nowadays face an ever-increasing number of options to choose from in a wide variety of settings. Besides, research on behavioral judgment and decision making has revealed that aspects of the choice set, such as the size of the set or complexity of it, may affect behavior (Schwartz, 2004). Therefore, in order to move the field forward, we suggest that future studies should investigate the effects of different nudges in more realistic and complex situations, while systematically taking into account the number of options in the immediate environment of the decision maker.

In the current studies, we did not find a moderating role of the number of options on the effectiveness of nudging on food choice. Given the current results, we can thus conclude that the proximity effect remains effective if there are less than 10 options in a choice set. However, it is important to highlight that in relation to research on choice overload, having nine options in a choice set may still be on the lower side of the spectrum (Chernev et al., 2015; Reutskaja et al., 2018) and may in fact be about the right number of options for some consumer products (Shah and Wolford, 2007). It remains to be determined whether the effect of nudges is also unaffected by the number of options when the size of the choice set increases further beyond the nine options presented in the current studies. In addition, the chocolates used in the current studies differed from each other in small and rather trivial dimensions, such as color of the wrapper and shape of the chocolate. The difference in taste between the different chocolates was unknown to many of the participants. Yet, choice complexity not only increases with the number of options, but also with the number of attributes that belong to each option (Chernev et al., 2015). To illustrate, the choice for a specific electronic device over another device can be based on a combination of specifications, such as battery life, memory, and processor speed, thereby complicating the decision. Therefore, while the current operationalization presented participants to a more challenging situation than in other studies of the proximity effect, it is important to stress that it is still not indicative of extremely complex decisions. Future research is required to test whether the level of complexity of choices moderates the effectiveness of nudging interventions in more extreme situations. In line with research on choice overload (Chernev et al., 2015) we would expect the effect of the nudge to become even more pronounced in these highly complex situations.

A major limitation of the current studies was that it was not feasible to vary the target chocolate across participants. Yet, it should also be noted that all options in the current experiment had the same utility. Apart from subjective preferences, which we tried to rule out as much as possible, none of the options was objectively superior or inferior to the other in order to simplify experimental control. This also implies that, in the current set of studies, we were strictly speaking about not “nudging for good”, but rather stimulating the selection of one alternative over a variety of others. Whether or not the intention of the nudge aligned with the nudgee’s goals, and how this altogether may impact the effectiveness of the nudge, was therefore beyond the scope of the current studies. The results of the current studies therefore imply that proximity can be effective in stimulating a specific option of a choice set with the same utility, but care should be taken into translating these findings, especially in regard to stimulation of desirable behavior. For example, in the context of health promotion, it will be relevant to examine whether proximity can effectively nudge the selection of healthy food over a variety of unhealthier alternatives.

Future research could dive deeper in the effectiveness of nudges in the face of an overload of options. An intriguing possibility remains that nudging may in fact be especially effective in promoting specific behavior in such circumstances. Not only is there room for improvement considering that people may make suboptimal decisions in such circumstances (Schwartz, 2004), there may also be something inherent to nudging that prevails in such circumstances: when people do not have the motivation or capacity to make optimal decisions. Moreover, nudges are most effective in the absence of clear preferences (Venema, 2020), and choice overload is most likely to occur when there is no dominant option for which one has a preference (Chernev et al., 2015). An interesting possibility is thus that nudges are most effective when choice situations become very complex as in people having no clear preferences because of being confronted with (too) many options. In such situations, nudges may facilitate decision making and guide decision makers effectively through this complex situation. Therefore, future research should investigate the effectiveness of nudging in more complicated situations.

Moreover, in the current studies we explored effects of the proximity nudge on subjective experiences during the decision making process. Future research can delve deeper into this by examining affective states prior to, during, and after making a decision. As nudges are intended to make the desirable option the easier choice, it would be interesting to examine whether this proposition is reflected in affective states as experienced by the decision maker. Moreover, better insights into the working mechanisms and boundary conditions of nudges and the proximity effect are required, not only for scientific progress, but also for practical and effective implementation in daily life. Current evidence seems to suggest that the proximity effect is driven by a decrease in perceived (physical) effort to obtain a particular option on offer, rather than by an increase in salience of that proximal option (Maas et al., 2012). Besides, it has been shown that the sight of proximal food activates eating-related cognitions and motor responses, thereby allowing for immediate interaction with the presented food (Junghans et al., 2013). The present studies add that this effortless route to the most proximal option is not affected by the number of alternative options. However, a coherent or conclusive explanation for the proximity effect is still required. Overall, the findings of the current studies suggest that the proximity effect can effectively steer food choice in a realistic and complex situation, regardless of whether the choice set is small or moderate in size.

## Data Availability Statement

The datasets generated for this study are available on request to the corresponding author.

## Ethics Statement

The studies involving human participants were reviewed and approved by the Ethics Committee of the Faculty of Social and Behavioural Sciences of Utrecht University. Written informed consent for participation was not required for this study in accordance with the national legislation and the institutional requirements.

## Author Contributions

LG, MA, and DR contributed to the conception and design of the study. LG performed the data analysis and wrote a first draft of the manuscript. MA and DR provided substantial comments and suggestions for improvement.

## Conflict of Interest

The authors declare that the research was conducted in the absence of any commercial or financial relationships that could be construed as a potential conflict of interest.

## Footnotes

^1^During the study, it was observed that some participants tasted the chocolate before filling out the question on satisfaction, while others did not. Therefore, results are not truly indicative of the satisfaction with the decision process, so results will not be reported in the results section. No significant results were found for this dependent variable.

^2^Again, results for satisfaction will not be reported in the results section for the same reason as in Study 1. However, in Study 2 results indicated a main effect of the number of options on satisfaction, *F*(1, 402) = 9.07, *p* = 0.003, *η_p_*2 = 0.02, such that participants were more satisfied when offered nine options (*M* = 4.99, *SD* = 1.78) than when offered three options (*M* = 4.44, *SD* = 1.95).
